# A comprehensive perspective of the innovation and reflection on virtual reality in the teaching and learning of anesthesiology

**DOI:** 10.3389/fmed.2025.1626292

**Published:** 2025-11-26

**Authors:** Jinzhi Zhang, Tairan Lin, Yu Shi, Yalin Wu, Fei Yu

**Affiliations:** 1 Peking University Shenzhen Hospital, Shenzhen, China; 2 Shenzhen Second People’s Hospital, The First Affiliated Hospital of Shenzhen University, Shenzhen, China

**Keywords:** virtual reality, anesthesiology teaching, innovative education model, comprehensive perspective, innovation and reflection

## Abstract

Virtual reality (VR) is a widely used tool in anesthesia education. By integrating multimedia and simulation technologies, it generates immersive environments that can serve as a controlled alternative to real-world training, thereby enhancing both theoretical knowledge and practical skills among anesthesia students. As a valuable, scientifically grounded, and feasible educational approach, VR helps foster student engagement and self-directed learning in anesthesiology. Additionally, it supports the development of more harmonious doctor–patient relationships and promotes standardized training and ethical practices in anesthesia. Given these advantages, the innovative application of VR in anesthesia teaching merits further exploration. In this article, an in-house VR system with independent intellectual property was developed at the author’s institution. This system is equipped with dedicated training modules designed for medical interns and graduate students.

## 1 Introduction

Virtual reality (VR) is a technology that integrates multimedia and simulation to generate visual, auditory, and tactile virtual scenarios resembling real environments. It allows users to interact with objects within these scenarios, producing an immersive sensation and experience. VR has extended Medical Extended Reality (MXR) in the medical field, which can be effectively applied through virtual reality, augmented reality, and mixed reality technologies (1). With the development of modern medicine, significant progress has been made in anesthesia technology, and its teaching mode has also been continuously improved with the advancement of technology, achieving favorable results (2). Especially in recent years, the development of VR has provided novel insights into the establishment of anesthesia teaching models (3). At the author’s institution, a VR system with independent intellectual property has been developed, complete with dedicated training facilities. This system has been utilized in the instruction and training of clinicians and medical students across various disciplines, demonstrating promising results. This article aims to comprehensively analyze the opportunities and challenges in anesthesia education based on the author’s firsthand experience. Furthermore, it discusses novel VR-based teaching models in anesthesiology. The findings are intended to offer practical insights and reflections to support the reform and innovation of anesthesia education.

## 2 Review of the current state of VR technology and its benefit in anesthesiology and its education

In medical training programs, teaching resources are often limited, as instructors primarily consist of practicing clinicians, and institutional educational funding is often constrained. These limitations can hinder the development of clinical skills among medical students and trainees, posing challenges for hospital education departments. To eliminate the shortcomings in the training process of spinal anesthesia, some researchers have adopted MIMICS, 3Ds MAX, and UNITY 3D to construct a VR and tactile feedback technology combined lumbar epidural anesthesia teaching platform based on the lumbar CT and MRI data of standard male volunteers. It has been found that this platform can effectively enhance the basic lumbar puncture ability of anesthesia interns (4). The use of tactile VR simulators to provide immersive guidance and training has a promoting effect on the skill training of clinical students in anesthesia of the inferior alveolar nerve. These students are provided with conditions for practicing inferior alveolar nerve blocks under near-real conditions and demonstrate higher accuracy and confidence (5). Chuan et al. prepared a VR trainer using high-resolution motion capture ultrasound images to train medical personnel in cognitive motor acupuncture skills required for ultrasound-guided regional anesthesia. The realistic operating environment provided by the software can simulate procedures under real conditions. As the experience of clinicians increases, the effectiveness of the software becomes more convincible (6). The VR simulation model of spinal ultrasound also has a similar effect. Through this model, anesthesia students without experience in spinal ultrasound imaging were trained, and their mastery of spinal ultrasound anatomy was tested before and after the training. It was found that after 1 h of self-study, the test scores of these students increased by 40% (7). Nurses in the operating room are assistants to anesthesiologists in the perioperative period. They can provide scenario simulation teaching to nurse interns in the operating room under the background of VR, resulting in improved theoretical and simulation performance and higher student satisfaction (8).

**Figure 1:**
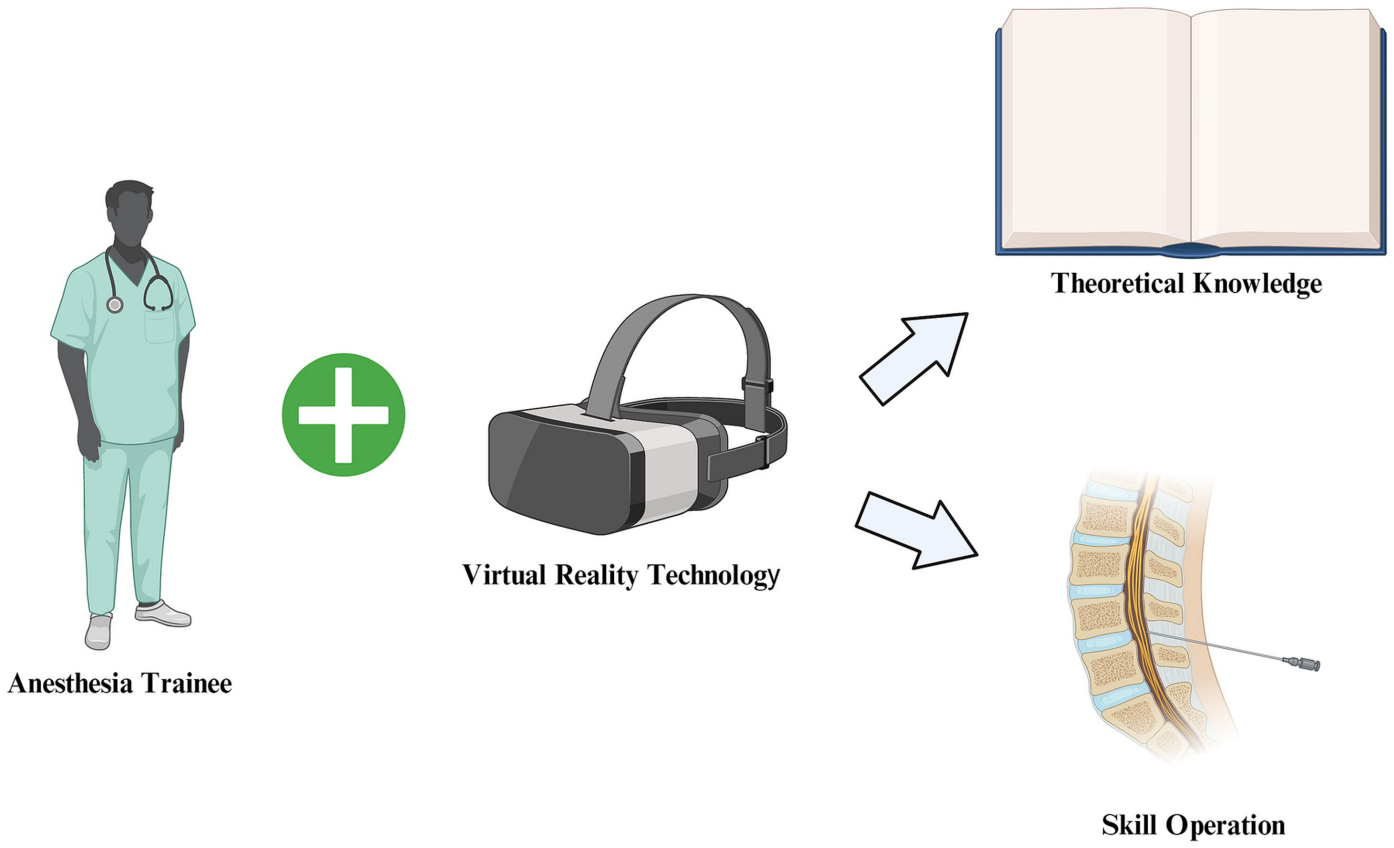
The application of VR in anesthesia teaching. Created with BioRender.com.

Anesthesiology is a critical subspecialty within clinical medicine. Becoming a qualified anesthesiologist requires not only knowledge of anatomy, physiology, and pathology but also proficient hands-on skills to apply clinical knowledge to perioperative anesthesia care. Traditional didactic teaching and simplified manual simulations often fail to achieve satisfactory educational outcomes. With advances in VR technology, head-mounted immersive learning systems and computer-based VR platforms can now provide anesthesia students with highly realistic procedural environments and more effective forms of skill retention. These systems support comprehensive training at both systemic and operational levels, using scenarios that closely mirror real clinical situations. They allow simulation of anesthesia procedures at varying difficulty levels and integrate teaching into vivid, real-world contexts. Through VR, students can achieve a deeper understanding of both theoretical principles and clinical techniques. As a result, students, clinicians, and nursing staff can all achieve favorable learning outcomes, explaining the growing adoption of VR in clinical training in recent years (Figure 1).

## 3 Buidling and set-up of the VR training facility in anesthesiology education

### 3.1 The necessity of integrating VR into anesthesia simulation teaching and practical skill training

Technological advancements have also promoted the establishment of medical education models. Especially in emergencies, such as the epidemic stage of coronavirus disease 2019 (COVID-19), it is also necessary to modify education models with the aid of VR. At present, distance learning is a commonly used learning technology in the field of anesthesia teaching, and it is of great importance for the education of anesthesiology, especially during the pandemic of COVID-19 when people cannot gather in public places (9). The application of VR and augmented reality (AR) has expanded beyond conventional simulation laboratories, and their training for medical personnel is not limited by geographical location. They have already been successfully applied to resuscitation, communication, and bronchoscopy training, which promotes the dissemination of anatomical knowledge such as diagnostic imaging (10). VR plays an important role in the education of clinical and nursing students. Through this technology, the satisfaction, self-efficacy, learning participation, and learning experience of students in clinical knowledge learning, basic operational skills, medical history inquiry, and communication skills can be significantly improved, indicating that it is an effective educational tool (11). Moreover, VR can also enable medical students to empathize and establish an identity exchange experience that resonates with patients, such as age-related macular degeneration, high-frequency hearing loss, and vision loss. On that basis, medical students can experience the pain of patients, understand the condition of patients from a local perspective, and cultivate empathy (12), thus contributing to communication with patients in clinical work and improving their humanistic literacy. Furthermore, VR can provide experiential learning that is replicable and repeatable, which is optimally cost-effective for medical training with specific learning objectives (13). Compared with conventional teaching methods, VR can also provide standardized training modes, ensuring the quality and effectiveness of education while shortening the learning curve of learners (14).

**Figure 2:**
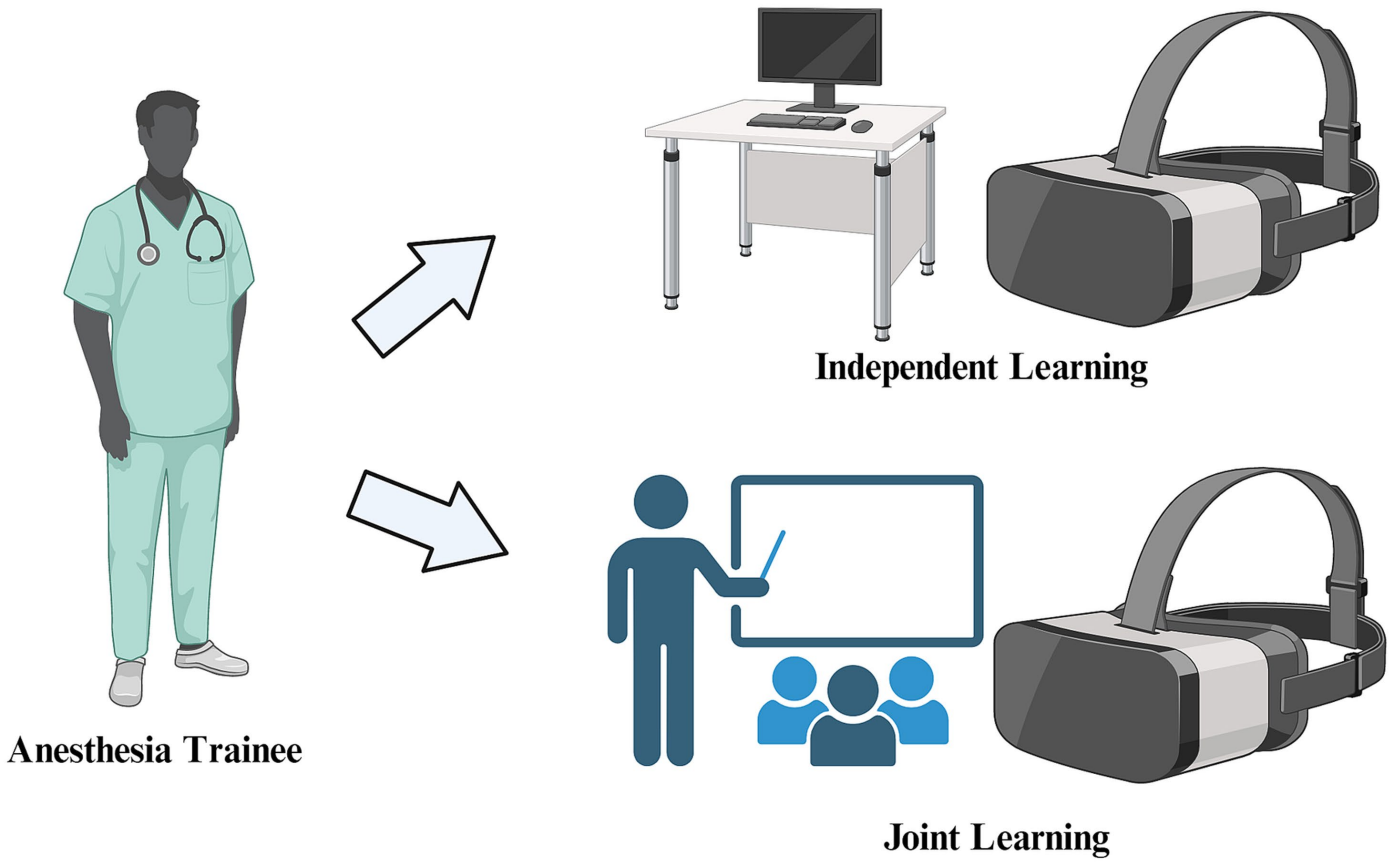
The advantages of VR in anesthesia teaching. Created with BioRender.com.

Therefore, VR holds essential value in anesthesia education. It can be used to develop standardized training courses that are easier to maintain and update, requiring less cost and space than traditional models. Unlike physical training models, which may show wear, VR-based scenarios can be reused consistently. VR training is also not confined to specific training sites; it supports both immersive sessions in equipped facilities and remote learning via network technology, mitigating disruptions caused by emergencies such as the pandemic (Figure 2). Furthermore, VR can simulate various stages of anesthesia training—from theoretical instruction and skill practice to preoperative visits and postoperative follow-up. It even allows students to experience patient perspectives during diagnostic and surgical processes. By enhancing the overall quality of clinical anesthesia teaching and reducing the time and cost required for individual skill acquisition, VR serves as a comprehensive educational aid.

### 3.2 The scientificity of integrating VR into anesthesia simulation teaching and skill training

In the anesthesia teaching process, there are some ethical and moral limitations, such as the need for certain procedures on corpses, animals, or special equipment (15). Conventional training models may reduce the number of samples available for students due to animal welfare and medical ethics reasons, and even some procedures can only be taught by the instructor and cannot be practiced in person. VR can effectively avoid these limitations by completely replicating various teaching models in a 1:1 ratio, thus allowing students to communicate with simulated real people in various realistic environments. In the scenarios constructed by VR, some students can realize that the visualized scenarios are not real but generated by computers, while others can fully reflect the scenarios in our daily lives (constructed from spherical or 360-degree videos and images). Of course, the two modes can also be combined under certain circumstances (16). In essence, this is involved in a method of constructing realistic scenarios according to the actual needs of students, which is much more scientific than the situation in conventional education where only a small number of standardized models can be purchased. Moreover, an increasing number of studies have confirmed that compared with conventional teaching methods, VR can improve the teaching quality of medical disciplines, and it is recommended that medical colleges should use this technology frequently (17). The advantage of VR also comes from the scientific nature of relevant systems. Many VR systems include both hardware and software. The hardware includes head-mounted devices, positioning devices, human teaching aids, servers, and control handles. The software is composed of the patient end, medical end, family end, and management end (18). The overall facility contains the most advanced knowledge and teaching aids, which also avoids the teaching quality bias caused by teachers’ personal knowledge reserves in conventional teaching.

In summary, VR can incorporate the most current and comprehensive knowledge systems within a scientifically structured framework, creating simulation scenarios that closely resemble—or even surpass—real clinical situations. This technology also helps bypass ethical and practical restrictions, freeing anesthesia students from regulatory and logistical constraints in their learning process. Instructors can focus more on teaching rather than on tedious lesson preparation or sourcing practical samples. As a novel medium of instruction, VR transforms traditionally didactic teaching into an engaging educational experience, ultimately enhancing the quality of education in this discipline.

### 3.3 Feasibility of integrating VR into anesthesia simulation teaching and skill training

With the advancement of technology, significant progress has also been made in VR, and the materials used in clinical medical training are mostly derived from actual situations, which achieves the traceability of the special scenarios in teaching and training. Taubert et al. captured videos using a 360-degree camera and used them as the real environment that medical students could experience in VR devices, thus providing their experience in radiation treatment from the perspective of patients. Students can learn more knowledge from VR videos than from daily lectures (19). Moreover, the learning conditions generated by VR are not affected by the human environment. Le Duff et al. applied a non-technical skill training system to scrubbing nurses based on VR environments and scenarios. They found that there was no difference in the acceptance level among medical personnel in France and Japan (20). Meanwhile, the advantage of VR lies in its ability to approach real-life situations without being affected by actual patient emotional changes. Some schools have adopted VR to provide basic operating room knowledge and skills for podiatry-related students, thus allowing them to learn and experience in a less stressful environment and improving their ability without posing excessive risks to patients (21). VR can also be integrated with other innovative technologies such as 3D printing to construct educational and training platforms for the learning of physical organs and systems, thus promoting their application among medical students and resident physicians (22). Some researchers have also developed a platform for VR scenarios through flexible user interaction and rendering of graphics processing unit textures involving multiple objects. The effectiveness of this approach has been verified in transcatheter heart stent implantation surgery, and preliminary results have been obtained (23).

Therefore, VR scenarios are grounded in reality: video materials can be recorded in clinical settings, and modeling data can be sourced from objective measures—such as medical imaging and physiological indicators from healthy volunteers or patients with relevant conditions. This ensures that VR-reconstructed scenarios are both realistic and reliable. Furthermore, by combining VR with other technologies—such as AR, 3D printing, and open R&D platforms—it is possible to create training environments that exceed what is feasible in real life. This versatile technology also supports the development of standardized training processes for anesthesia students, residents, specialists, and nursing staff. Such standardization enables consistent training across different regions and populations. Trainees can engage with the technology more confidently due to its high fault tolerance and low risks. These attributes collectively enhance the feasibility of applying VR to simulation teaching and skill training in anesthesiology.

### 3.4 The VR system developed by our hospital and its future application in anesthesia teaching

Supported by the Shenzhen National Industrial Base Public Service Platform Project, our hospital has obtained funding under the Shenzhen Virtual Reality Clinical Application Public Service Platform Project to procure a range of VR equipment, including spherical panoramic cameras, image data workstations, motion optical tracking systems, electromagnetic positioning tracking systems, VR headsets and controllers, VR gloves, MR glasses, VR all-in-one machines, graphics workstations, cloud servers and networking infrastructure, and physiological simulators. A 1,600 m^2^ VR teaching and training center has been established within the hospital.

A ultrasound image integrated interactive display system with independent intellectual property rights, comprising multiple modules—human simulation, pose fusion calculation, and ultrasound interactive display units, has been developed by our hospital, which enables ttrainees to perform realistic hands-on practice in an interactive virtual environment. Furthermore, a VR ultrasound image simulation training system has been built based on a 5G cloud network architecture for medical ultrasound teaching and application data exchange. This system employs a 5G industry virtual private network architecture, substantially reducing enterprise network deployment costs. It also incorporates a 5G air interface latency reduction solution and utilizes a municipal 5G-LAN architecture to achieve local data offloading and processing. Leveraging 5G edge computing capabilities, the system enables low-latency interaction between lightweight terminals and the 5G edge cloud platform, thereby realizing an integrated virtual-real presentation. Currently, multiple articles have been published for the project (24, 25), and we have applied for invention patents for this project (Table 1), exhibiting great pioneering significance in China. Although the VR system was initially applied in ultrasound medical education, its scalable instructional framework holds reference value for other disciplines. In the future, it could be extended to anesthesia teaching. Relying on the VR training center and R&D team, we plan to develop VR-based anesthesia teaching scenarios and simulators, compile VR teaching materials and training plans, and establish standardized operating procedures (SOPs) for VR-assisted anesthesia education. These resources will support the training of undergraduates, postgraduates, resident physicians, specialist trainees, and advanced practitioners in anesthesiology.

**Table 1:** Patents related to virtual reality technology produced by our hospital or collaborate with other institutions.

Patent number	Patent content	References
1	A method for generating 3D point cloud models from ultrasound images based on deep learning	(30)
2	Facial expression recognition methods, devices, computer equipment, and computer-readable storage media	(31)
3	Image recognition methods, devices, computer equipment, and computer-readable storage media	(32)
4	A language recognition method and device	(33)
5	A multi module integrated interactive display system for ultrasound imaging	(34)
6	A medical ultrasound simulation teaching method and system	(35)

In China, there are few VR training systems with independent intellectual property. The system developed by our hospital is expected to address the growing teaching and training needs of anesthesia students. It also offers economic benefits by transforming the training model for anesthesiologists at our hospital and facilitating the efficient cultivation of high-quality clinical talent. Moreover, the system holds social value: the VR training courses and SOPs developed for anesthesia education could be promoted across Shenzhen, Guangdong Province, and even nationwide, thereby supporting the reform of anesthesia teaching in China. Ultimately, the adoption of this system is expected to significantly reduce training costs for anesthesia students and contribute to the advancement of anesthesiology.

## 4 Current and future projects for the VR training facility in anesthesia teaching

### 4.1 VR stimulates the enthusiasm and self-discipline of anesthesia teachers and students

Conventional medical education is usually implemented through imparting textbook knowledge, clinical internships, and clinical practice, interspersed with standardized models or patient teaching models. Teachers need to spend much time preparing lessons, formulating lesson plans, writing slides or blackboards, and improving the vividness of their courses, so as to assist students in remembering more knowledge or achieving better grades. In this process, students can master various knowledge points by rote through pre-class preparation, classroom listening, and post-class review. Besides, students with more flexible thinking may combine image pictures from the internet to consolidate their memory of these knowledge points. However, conventional medical education methods have achieved half the results with twice the effort from the perspective of both teachers and students, resulting in low teaching quality and insufficient learning interest, which is not conducive to the reserve of clinical knowledge (17).

After the emergence of VR, learning can become an exciting and vivid experience for students, and self-regulation in the learning process can also be generated. After the application of immersive VR experience learning, the analysis of students will be more organized, and more reflective conclusions can be drawn (26). Memory research has also corroborated that VR experiences can make knowledge more vivid in the memory process by simulating the multimodality, vividness, and inclusiveness of life experiences, thus making it easier for participants to remember through the knowledge itself. The electrophysiological phenomena behind memory also indicate that VR can enable participants to experience even relaxed memory processes (27). Teachers have also established more real models based on VR, and they can select real models as samples in their teaching process to more vividly convey their ideas. In addition, students can learn independently; teachers can pay more attention to more cutting-edge knowledge or knowledge explanations, thus avoiding the consumption of time for preparing non-knowledge content in conventional teaching. The time saved for teachers can be used for new knowledge reserves or self-adjustment of emotions, and they will also be more passionate about teaching work, thus forming a virtuous cycle. Especially for anesthesiologists, regardless of students or teachers, VR can enhance their experience and improve their efficiency in learning and teaching. Therefore, VR can enhance the enthusiasm and self-discipline of anesthesia teachers and students.

### 4.2 VR promotes the standardization of anesthesia education ethics and harmonizes doctor–patient relationships

There are inevitable moral and ethical issues in medical education (28), such as how to use the most beneficial and least harmful procedures to alleviate the pain of patients, how to show maximum respect to unconscious patients, how to provide patients during palliative care with the right to choose their treatment methods, and how to legally obtain the human samples needed by medical students.

As mentioned earlier, in VR, anesthesiologists can exchange identities with various patients, empathize with their misfortunes, understand their patients, and provide the most beneficial treatment. With the same perception as patients, anesthesiologists can better communicate with them to understand their thoughts and needs. Under this training mode, anesthesiologists can better adhere to their original intentions and empathize with patients, thus establishing a harmonious relationship with patients (12).

Moreover, the application of VR also reduces the psychological pressure and burden on students, as they are facing “real patients” generated by machines and there are remedial measures for some dangerous operations (29). This enables students to become qualified anesthesiologists based on these lessons and avoids medical accidents in real diagnosis and treatment environments. Meanwhile, it is not necessary to consider the legality of sources or the availability of samples for various scenarios and samples simulated by VR. Anesthesiologists can better practice their operations and cultivate more outstanding anesthesiologists.

However, as VR technology evolves, it is essential to establish clear regulations and guidelines governing its use in anesthesia education and training. Such frameworks can help prevent unnecessary breaches of patient or student privacy, curb overreliance on VR in medical instruction, and ensure that anesthesiologists develop both independent clinical judgment and excellent technical competence.

## 5 Teaching and learning pedagogical framework of anesthesiology using VR

The educational objectives of using VR in anesthesiology is improve the teaching quality of teachers and the learning quality of students, shorten teaching and learning time, improve teaching and learning efficiency, and reduce teaching and learning costs. The learners include undergraduate students (in the stage of theoretical learning and clinical internship), master’s students or resident physician standardized training students (in the stage of clinical learning), and advanced doctors (in the stage of senior resident physicians, junior attending physicians, or other doctors with special skill learning needs). The teachers include anesthesiologists with teaching qualifications (including theoretical teaching and clinical practice teaching). The methods of skill evaluation and feedback include theoretical study paper answer scores, accuracy of case analysis, proficiency in clinical practice learning skills, effective information obtained from communication between standardized patients and learners, and the satisfaction level of teachers and students towards themselves and each other.

## 6 Conclusion

We should also critically recognize the application of virtual reality technology in anesthesia teaching. There are also certain limitations and risks in this field, such as the reduction of interpersonal interaction in anesthesia teaching activities by virtual reality technology. This technology sacrifices the judgment of clinical doctors and teachers, leading to excessive reliance on technology in anesthesia teaching. Potential ethical issues and side effects (such as network diseases) should also be taken seriously and better solutions proposed. In our article, there is also a lack of specific comparisons between virtual reality technology and other learning methods, such as the objective effects that virtual reality technology brings to anesthesia students’ skill learning compared to traditional methods. Currently, there is relatively little research on this topic, which is also the research content that our team needs to continue to carry out in the future.

However, the development of science and technology has provided novel concepts and models for anesthesia education. Virtual reality (VR) is widely used in anesthesia teaching and research at various levels due to its necessity, scientificity, and feasibility in anesthesia teaching reform. This technology can not only stimulate the enthusiasm and self-discipline of anesthesia students but also promote harmonious doctor–patient relationships, thus contributing to more standardized anesthesia education ethics and the improvement of regulations and rules. Based on the practice of VR in the teaching field in our institute and the above analysis, it can be maintained that hospitals and universities can use VR to strengthen the training of anesthesia students, shorten their training curve, and improve the training quality. These efforts are expected to provide solid support for anesthesia students to respond to opportunities and challenges in the medical field, thus contributing to human health.
